# The effect of developmental temperature and inbreeding on sexual and reproductive traits of female guppies

**DOI:** 10.1093/beheco/arag101

**Published:** 2026-08-26

**Authors:** Md Mahmud-Al-Hasan, Michael D Jennions, Megan L Head

**Affiliations:** Division of Ecology and Evolution, Research School of Biology, Australian National University, 46 Sullivans Creek Road, Canberra, Australian Capital Territory 2600, Australia; Division of Ecology and Evolution, Research School of Biology, Australian National University, 46 Sullivans Creek Road, Canberra, Australian Capital Territory 2600, Australia; Stellenbosch Institute for Advanced Study (STIAS), Wallenberg Centre, 10 Marais Street, Stellenbosch 7600, South Africa; Division of Ecology and Evolution, Research School of Biology, Australian National University, 46 Sullivans Creek Road, Canberra, Australian Capital Territory 2600, Australia

**Keywords:** female attractiveness, male mate choice, developmental temperature, inbreeding, *Poecilia reticulata*, sexual selection

## Abstract

Global temperatures are rising and inbreeding is increasingly common in wild populations as habitats become fragmented and population sizes shrink. There is extensive research on how inbreeding or elevated temperatures affect animal performance, but little is known about how these 2 factors interact to affect sexually selected behavior and reproductive traits. We ran a 2 × 2 experiment to investigate how developmental temperature (26 °C or 30 °C) and inbreeding (outbreeding or full-sibling mating) affect female guppies (*Poecilia reticulata*). We reciprocally cross-bred full-siblings to create inbred and outbred females and measured their sexual attractiveness (via male mate choice tests), fecundity, egg diameter and total egg mass (*N* = 120 females). Female attractiveness was affected by an interaction between breeding status and temperature. For females reared at 26 °C, males preferred outbred females over inbred females and over both inbred and outbred females raised at 30 °C. In contrast, for females reared at 30 °C, males did not prefer outbred females. Inbreeding and elevated developmental temperature independently reduced egg diameter, indicating that both factors reduce offspring provisioning. Neither inbreeding nor developmental temperature affected fecundity or total egg mass. However, larger and older females produced more eggs, and larger females had heavier clutches. Our results suggest that the fitness effect of inbreeding for females depends on developmental temperature. Climate warming may intensify these genotype–environment interactions thereby increasing the costs of inbreeding. The efficacy of sexual selection in removing deleterious recessive alleles could be reduced if males are less likely to avoid inbred females as temperatures rise.

## Teaser

Inbreeding and high temperatures can reduce animal performance, but how do they combine to affect mating and reproductive success? We raised inbred and outbred female guppies at normal or warm temperatures. Inbreeding and warming reduced egg size but not egg number. Males preferred outbreds when females developed at normal temperatures, but this preference disappeared when females developed at warmer temperatures. These findings suggest that climate warming may change how inbreeding affects reproduction and mate choice.

## Introduction

Climate change is a major concern as mean temperatures continue to rise globally. Extremes in temperature can seriously damage organismal performance (Wegner et al. 2008). High temperatures are a major challenge for poikilothermic organisms because metabolism accelerates when the temperature rises (Angilletta et al. 2004). Animals die if they cannot maintain metabolic homeostasis. As a result, unusually high (or low) temperatures can lead to a decline in population size if they lower female reproductive output. Smaller populations can, in turn, create additional problems for species persistence because of an increase in inbreeding, which can cause inbreeding depression (Keller and Waller 2002; Crnokrak and Roff 1999). Inbreeding depression is a well-documented decline in the expression of fitness-enhancing traits by inbred individuals. Inbreeding increases homozygosity which leads to deleterious recessive alleles more often affecting phenotypes because they are not masked by dominant alleles (Hare et al. 2011). This begs a question: do higher temperatures and inbreeding interact to exacerbate harm to females and thereby accelerate a decline in population size?

Both high temperatures during development and inbreeding can negatively affect reproductive performance and mate choice in animals (Keller and Waller 2002; Szulkin et al. 2013; Candolin 2019; Walsh et al. 2019). In ectotherms, elevated temperatures typically accelerate development but individuals mature at a smaller body size. This pattern is sufficiently common to be called the “temperature–size rule” (Angilletta et al. 2004). As a result, females reared at high temperatures are often less attractive to males (Silva et al. 2007). High temperatures can also disrupt oogenesis and vitellogenesis, thereby reducing maternal provisioning (eg, in fishes: Angilletta et al. 2004; Angilletta and Dunham 2003; Pankhurst and Munday 2011; Van Der Kraak and Pankhurst 1997), resulting in females producing fewer or smaller eggs than those raised at normal temperatures (Pankhurst and Munday 2011). Similarly, numerous studies in many taxa have shown that inbred females have lower fecundity and produce less viable offspring (Crnokrak and Roff 1999; Keller and Waller 2002). In fishes, for example, being inbred is linked to smaller broods, smaller eggs, and poorer offspring performance (Van Oosterhout et al. 2007; Heath et al. 2002). Being inbred may similarly decrease female attractiveness to males, because inbreeding can reduce body size, sexual coloration, and body condition (Schlupp and Jia 2018). In guppies (*Poecilia reticulata*), for example, male mate choice is often based on condition-dependent cues which seem to be correlated with fecundity and maternal provisioning. Any temperature or inbreeding induced changes in size, condition, or sexual signaling may lower a female's attractiveness and reduce the mating effort that males will direct towards her (Herdman et al. 2004; O’Grady et al. 2006; Fox and Reed 2011; Schlupp and Jia 2018). Such shifts in male mate choice have implications for the efficacy of sexual selection in removing deleterious recessive alleles.

Despite strong evidence that temperature and inbreeding independently reduce female attractiveness and reproductive success, to our knowledge no experimental study on vertebrates has tested for their combined effects on females. Theory and meta-analyses of existing data suggest that inbreeding depression is often amplified under environmental stress. Stress can increase the phenotypic difference between the expression of deleterious recessive alleles in homozygotes and the expression of dominant alleles; and/or stress can limit the ability of compensatory physiological mechanisms to ameliorate developmental errors that arise from inbreeding (O’Grady et al. 2006; Fox and Reed 2011). There is some evidence that inbred populations experience disproportionately greater declines in reproductive performance when food is limited or temperatures are high (Kristensen et al. 2008; Fox and Reed 2011). To date, however, most empirical studies have investigated the effects of inbreeding and thermal stress separately. It remains unclear whether these stressors interact to affect female reproductive success. It is biologically relevant to test for such an interaction because thermal stress due to climate change and inbreeding due to population decline mean that both factors often act together in the wild.

Guppies (*Poecilia reticulata*) are an ideal model to test for the combined effects of inbreeding and high temperatures. They are small, live-bearing fish found in shallow tropical streams that often become isolated pools in the dry season (Griffiths and Magurran 1997). As such, mating with a relative is a common occurrence in the wild (Hain and Neff 2007). Inbreeding has negative effects on many guppy life history traits (see table 2 in Mahmud-Al-Hasan et al. 2025b). Further, females have been shown to adjust their maternal investment (eg brood and egg size) in response to prevailing environmental conditions (Reznick et al. 1996), including temperature (Herdman et al. 2004; Chung et al. 2026; Moura-Campos et al. 2025).

Male mate choice—though less studied than female choice—is well known in poeciliid fishes. Male guppies typically prefer females with traits that predict their reproductive output, such as female body size and condition, visual indicators of receptivity, olfactory signals, and behavioral responses to courtship, and adjust their mating effort accordingly (Herdman et al. 2004; Guevara-Fiore et al. 2010a; Schlupp and Jia 2018; Saoshiro and Karino 2023). Female cues that attract males are often condition-dependent, and body condition also affects a female's investment in fecundity, egg size, and offspring provisioning (Kamler 2005). The cues used by males to assess females can therefore serve as reliable indicators of their quality as a mate (Chambers and Leggett 1996; Kamler 2005). Although males cannot directly assess egg size or fecundity, they can use proxy cues that covary with maternal allocation and offspring quality, such as female size, body condition, and courtship responses, to choose high quality females (Cotton et al. 2004; Herdman et al. 2004). Because these cues are condition-dependent, any factor that reduces a female's condition, including inbreeding and elevated developmental temperature, should reduce her expression of the traits males assess, and therefore the mating effort males direct toward her. To our knowledge, however, no study has directly tested whether female inbreeding or developmental temperature alters a female's attractiveness to choosing males.

Here, we assess how inbreeding and developmental temperature affect the sexual and reproductive traits of female guppies. We used a fully balanced, controlled breeding design, to generate inbred and outbred females and reared them from birth until maturity at either a control (26 °C) or elevated temperature (30 °C). We then measured female attractiveness in male mate choice trials, fecundity (number of eggs), egg size, and total egg mass. We asked 3 key questions:

1. Does inbreeding lower female attractiveness and reduce their reproductive investment? Based on previous findings, we predicted that inbred females would be less attractive to males and would produce fewer and smaller eggs, with a lower total egg mass than outbred females (Crnokrak and Roff 1999; Van Oosterhout et al. 2007).
2. Does an elevated developmental temperature lower female attractiveness and reproductive investment? Based on the temperature–size rule, we predicted that females reared at 30 °C would be smaller, hence have lower fecundity and produce smaller eggs than females reared at 26 °C (Angilletta et al. 2004; Pankhurst and Munday 2011; Mahmud-Al-Hasan et al. 2025a).
3. Do inbreeding and developmental temperature interact? If these 2 stressors affect overlapping physiological pathways, we predicted a synergistic effect such that inbred females reared at a higher temperature have a disproportionate decline in attractiveness and reproductive output than is predicted by either factor acting on its own.

## Materials and methods

### Origin and maintenance of fish

We sourced our fish from a stock population that was established at the Australian National University in 2018 from 2 independent populations collected from Alligator Creek near Townsville (19.45°S, 146.97°E) in 2002 (Lindholm et al. 2014) and 2010 (Kranz et al. 2018). Our stock population had >200 individuals per generation. Each generation was reared in at least 4 different tanks, each with a maximum of 50 fish. Males and females were separated into different tanks before maturity so they remained virgin until we conducted controlled matings. Newborn fry were collected and reared in 3 L tanks (up to 10 fish per tank) under a 14:10 h photoperiod at 26 °C and fed ad libitum with *Artemia nauplii* twice daily.

### Establishing inbreeding and temperature treatment

We followed the same experimental design used by Mahmud-Al-Hasan et al. (2025a, 2026a). In brief, we created 150 male–female pairs of unrelated virgins. Each pair was housed in a 3 L plastic tank. These pairs generated 80 outbred F1 families of full-siblings. We only used pairs with 4 or more offspring. Pairs that produced fewer offspring, failed to breed in a timely fashion, or experienced early brood loss were not used. From each family we kept a maximum of 14 offspring. Keeping families with 4 to 14 offspring increased the likelihood that we could successfully rear 2 males and 2 females required per family to create 4 types of experimental crosses between 2 families. To create inbred and outbred offspring (F2) we used a fully balanced block design that involved mating individuals from 2 F1 families per block (eg, block 1: family A and B; block 2: family C and D; and so on). Brothers and sisters were paired in 3 L tanks to create pairs of related fish (AA and BB) and nonsiblings were reciprocally paired (male–female; female–male) to create pairs of unrelated fish (AB and BA). We created a total of 30 blocks using 60 families. Fish were maintained under the same conditions and feeding regime as described for the stock fish (see above). After 2 wk we removed the males from the tanks and monitored the females daily until they gave birth.

When females gave birth, we randomly collected 16 newborns from each of the 2 inbred and 2 outbred families in each block (*N* = 64 offspring/block). Offspring were photographed to measure their standard length (SL) and then individually placed in separate 1 L tanks. We then immediately assigned the tanks to 1 of 2 temperature-controlled rooms: 32 tanks were kept at a control temperature (26° ± 1 ° C) and the other 32 at a higher temperature (30° ± 1 ° C). Tanks were assigned so that for each set of 16 newborns, 8 offspring were reared at the control temperature and 8 offspring were reared at the high temperature. Fry cannot be sexed at birth, but by setting up so many offspring we (i) increased our chances of being able to set up females from each family at both developmental temperatures and (ii) obtained sufficient sexually mature females for the current study.

The room temperatures of 26 and 30 °C resulted in mean water temperatures of 24.4 ± 0.6 °C and 27.8 ± 0.4 °C, respectively. In a previous study using the same room-based rearing design, Chung et al. (2026) found no evidence that treatment differences were driven by room effects. These water temperatures match those experienced by wild guppies which typically range between 20 and 28 °C (Weather and Climate 2025), but can occasionally exceed 29 to 30 °C in shallow pools (Alkins-Koo 2000; Grether et al. 2001; Maharaj and Alkins-Koo 2007). The ∼3.4 °C water-temperature difference between the treatments reflects projected end-of-century warming under high-emission scenarios, where a 4 °C rise in air temperature is predicted to translate into a 3 to 3.5 °C rise in stream water temperature (Caissie et al. 2001; Meinshausen et al. 2020). Our design thus replicates both present-day extremes and anticipated future temperatures for wild guppies.

Starting from 4 wk after birth, we inspected the tanks daily to determine when females matured. Females were considered mature once we could see yolked eggs through the body wall (Moura-Campos et al. 2025). Two weeks after maturation, females from the 30 °C treatment were put back in the control temperature of 26 °C for at least another 2 wk (Fig. 1). This ensured a common garden temperature for all females prior to measuring their sexual and reproductive traits. This control for transient effects of recent temperature (see Le Roy et al. 2017) does, however, mean that females from the 30 °C experienced an unavoidable change in water temperature which females in the control treatment did not. Finally, 4 wk after maturation, females were available for us to conduct behavioral trials and measure reproductive traits.

**Figure 1:**
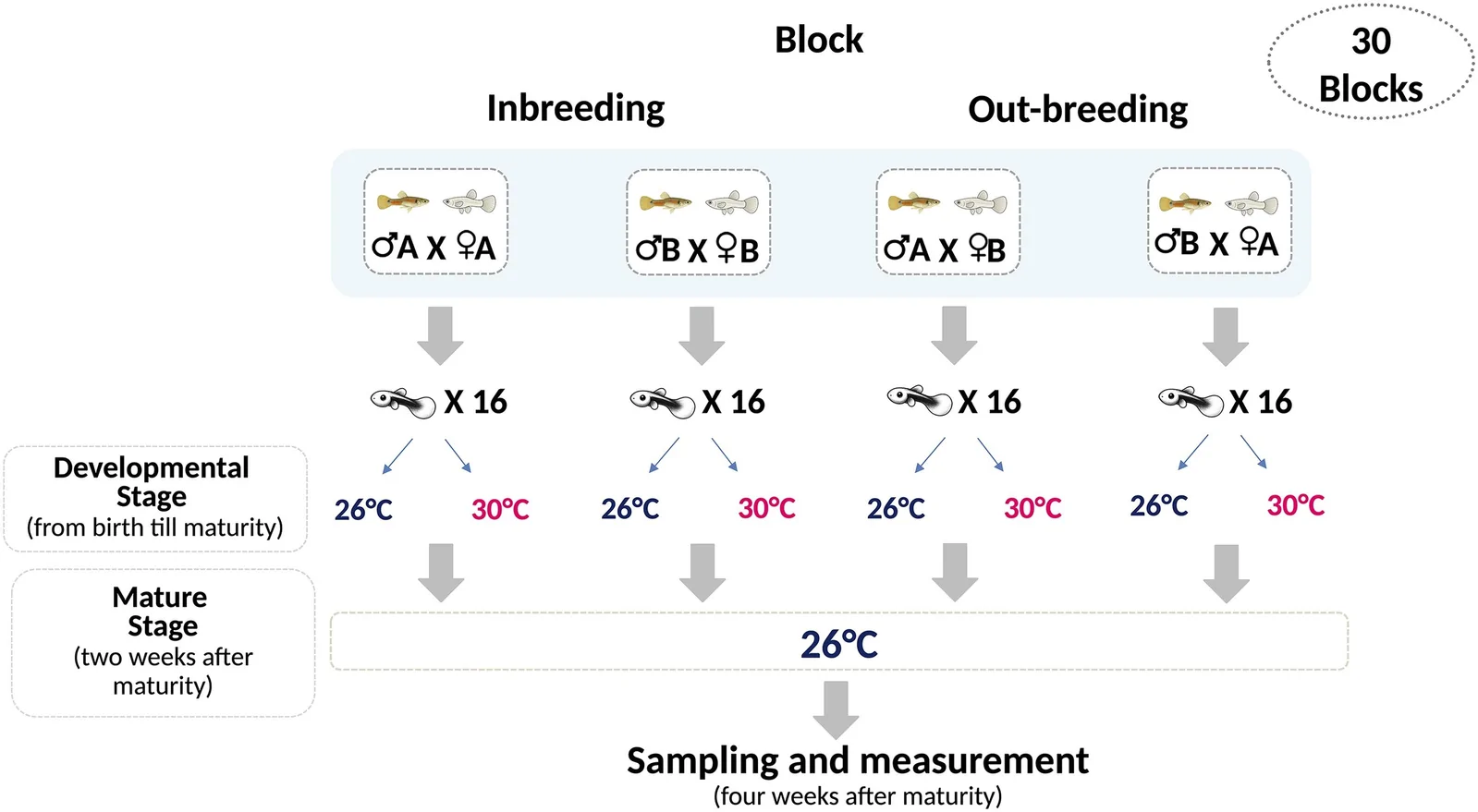
Schematic representation of the experimental design. Inbred and outbred fish were generated using a fully balanced block design. Females were reared at either 26 °C or 30 °C from birth until sexual maturity. All females were then kept at 26 °C for at least 2 wk before we measured their sexual and reproductive traits. The final sample size was *N* = 30 for all 4 treatment groups.

### Female attractiveness

In each mate choice trial, an adult, virgin stock male (SL, 16.71 ± 0.16 mm, mean ± SE; *n* = 30) was placed in a clear plastic cylinder in the center of a square tank (45 cm L × 45 cm W × 10 cm D). The tank had 4 corner compartments, each of which held a female from a different treatment group (inbred, control temperature, IC; inbred, hot temperature, IT; outbred, control temperature, OC; outbred, hot temperature, OT) from the same block (Fig. 2). The 4 females differed in their time to maturation, and we could only begin a trial once a female from each treatment type had reached maturity. Females were 29 to 71 d post-maturation when tested with all females (including those from the 30 °C treatment) maintained at 26 °C throughout this period until trials were conducted. We controlled statistically for the resultant variation in female age. Females were randomly assigned to a corner compartment, and the observer was blind to the treatment type. We used a new test male for each set of 4 females.

**Figure 2:**
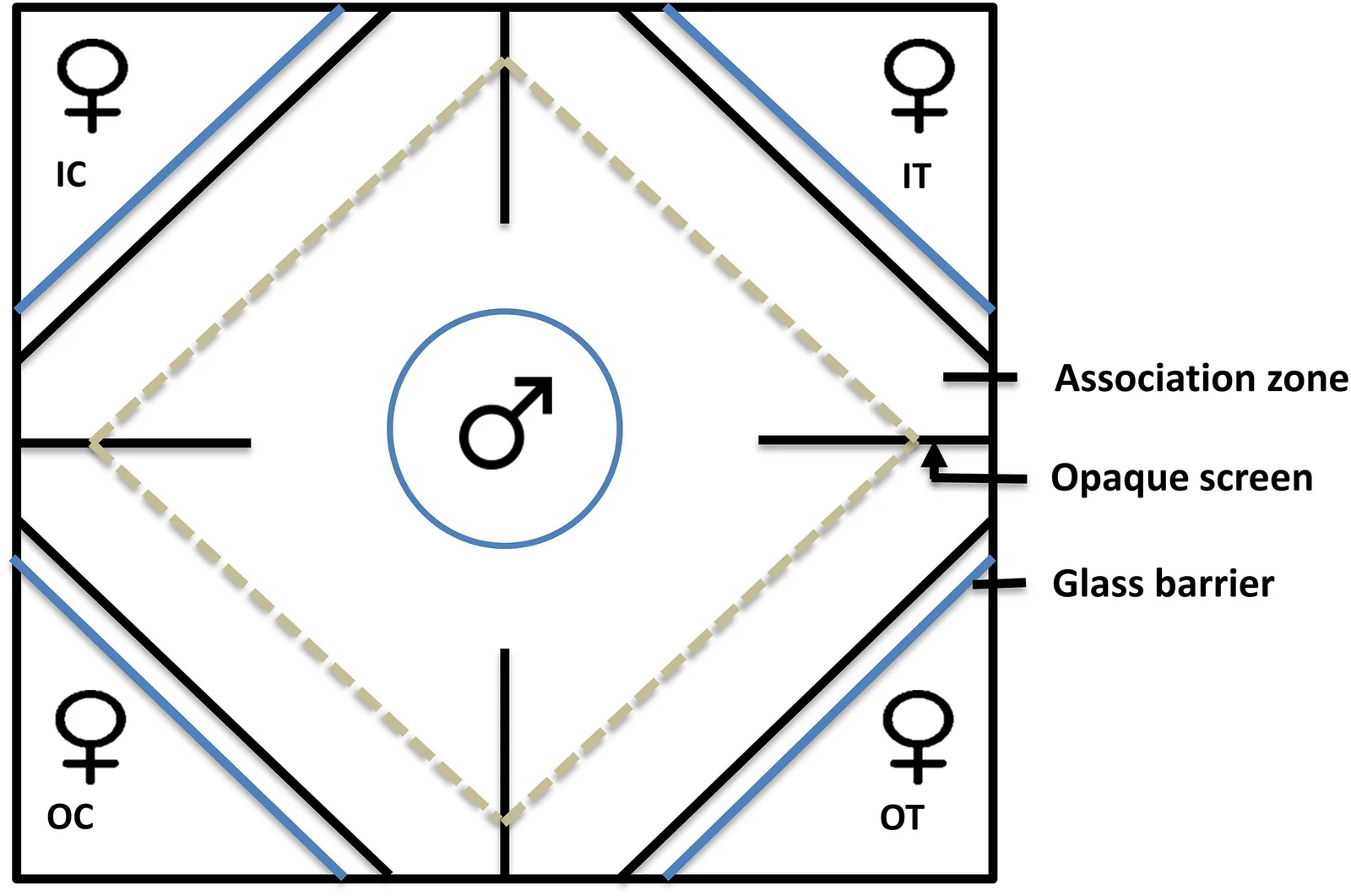
Schematic representation of the tank used for the female attractiveness trials.

Each corner compartment was separated from the central compartment by a removable opaque screen. Behind each opaque screen there was a transparent glass barrier to allow the male to see the females during the choice trial. Each wall of the central compartment had a protruding opaque screen to prevent visual contact between adjacent females during the trial. After a 5-min acclimation period, we removed the opaque barriers in the corners, and released the male from the central cylinder. We then video-recorded his association time with each of the 4 females for 15 min following Booksmythe et al. (2013). The male's preference for each female was quantified from video recordings as the time he spent <4 cm from the female's compartment (equivalent to approximately 2 body lengths of a male). To minimize carry-over cues, we changed the water between trials to remove potential olfactory cues. The location of females was randomized between trials.

### Female fecundity and egg traits

To measure reproductive allocation at a standardized time point, females were euthanized with *Aqui-S* (0.0075% v/v) immediately after the attractiveness trials and their SL (mm) was measured. We then dissected out eggs under a microscope. By using virgin females we avoided any confounding factors associated with measuring realized fecundity when recording live births (eg, paternal effects, effects of sexual harassment, gestation period, and embryo loss). We counted the number of eggs available for fertilization (ie female fecundity) and weighed them (‘total egg mass’). Immediately after dissection, the full clutch was transferred to a pre-tared weighing vessel, gently blotted to remove surface moisture using a fresh Kimwipe for each clutch, and weighed on an analytical balance. Weighing was completed within ∼20 s of dissection to minimize variation due to dehydration during handling. We also photographed eggs under a dissecting microscope alongside a reference scale, and measured the diameter of 5 randomly chosen eggs (repeatability of egg size among females: *r* = 0.47 ± 0.044 SE, 95% CI: 0.375 to 0.551, *P* < 0.05, df = 1, *n* = 120 females, 30 per treatment group) using *ImageJ* (Aich et al. 2020a).

### Statistical analysis

To test whether developmental temperature and inbreeding affected the sexual and reproductive traits of female guppies we ran generalized linear mixed-effect models (GLMMs). In all models, temperature (26 °C or 30 °C) and breeding status (inbred or outbred) and their interaction were treated as fixed factors; a female's paternal family identity was treated as a random factor to account for the measurement of several females from the same family, and block identity was treated as a random factor to account for repeated use of parents from 2 families per block (eg AA, AB, BA, and BB pairings) (for the equivalent analysis see: Vega-Trejo et al. 2016; Mahmud-Al-Hasan et al. 2025a, 2026a).

We ran separate GLMMs with Gaussian error distributions for 4 response variables: attractiveness (male association time in choice trials), total number of eggs, total egg mass, and average egg diameter.

For female attractiveness we used the time with each female as a data point and included female age (zero-centered for the 4 females per trial) as a covariate in the GLMM to take into account that her age when tested might affect her attractiveness. We included the male's identity as a random factor to account for variation among males in the average time they spent with females (ie there was data from 4 females per male/trial). On average, females spent <50% of each trial in the association zones near females and we therefore treat “time with each female” as 4 quasi-independent data points because time with 1 female does not automatically reduce the time spent with another female. Our approach does not give higher weighting to males that spent more time with females because we are interested in the order in which males rank females as mates rather than the time spent with them per se. Our statistical approach is a standard 1 used in many studies that involve choice between more than 2 potential mates (eg, Booksmythe et al. 2016; Aich et al. 2020b; Chung et al. 2024b). We also ran a second GLMM that included female body size (zero-centered for the 4 females per trial) as an additional fixed covariate (body size difference was weakly correlated with age difference in our sample of females, *r* = 0.36, see Materials). We did so because there was a difference in mean body size between inbred and outbred females in our sample (see Figure S4). This size difference was due to either chance when sub-sampling females or an effect of inbreeding on post-maturation growth. We say this because there was no effect of inbreeding on female size at maturity in a much larger sample of females which we subsampled for the current experiment (Mahmud-Al-Hasan et al. 2025a). For egg number, total egg mass and egg diameter we included female size and female age as fixed covariates in the initial GLMMs because of their well-known effects on reproductive traits (Karino and Ikeuchi 2011; Žák and Reichard 2021). We also ran GLMMs that excluded female size because it is an intermediate variable that might itself be affected by inbreeding and developmental temperature. Female age and female size were weakly correlated (*r* = 0.39), well below the level that would indicate problematic multicollinearity. We therefore retained both as covariates in the relevant models, as each captures a biologically distinct source of variation in reproductive traits. There was, however, no change in the effects of inbreeding and developmental temperature when female size was removed from models (see Supplementary Materials).

Repeatability of egg diameter was estimated in R using rptR (rptGaussian) by fitting a Gaussian mixed-effects model with female ID as a random intercept (egg diameter ∼1 + (1|female ID)). We used 600 egg measurements from 120 females; confidence intervals were obtained from 1,000 bootstraps and significance from 1,000 permutations.

We always included the interaction between breeding status and developmental temperature in initial models. If the interaction was not significant, we removed it from the final model to quantify main effects. Its removal did not significantly decrease the fit of any of the GLMMs (see Supplementary Materials).

Models were run using R v4.3.1 in Rstudio. We used the DHARMa package (Hartig 2016) to run model diagnostics. If the residuals were not normally distributed, we power-transformed the response variable to improve the fit to a Gaussian error distribution. Specifically, attractiveness, egg number, and mean egg diameter were power-transformed; total egg mass did not need to be transformed. We applied ANOVA type III Wald chi-square tests for fixed effects in models that included the breeding status by temperature interaction, and type II tests in models excluding the interaction. In all tests the level of significance was set at *α* = 0.05 (2-tailed). Summary statistics are presented as mean ± SE.

The project was approved by the ANU Animal Ethics Committee (A2021/04). Guppies are pests in Australia and cannot legally be released back into the wild. All fish were therefore euthanized on completion of the study. We therefore strove to collect data from as many fish as possible within given time constraints.

## Results

### Female attractiveness

Males spent an average of 43.6 ± 2.2% (mean ± SE) of their time associating with females. There was a significant interaction between inbreeding and developmental temperature that affected female attractiveness (Fig. 3). Inbreeding affected attractiveness for females reared at 26 °C, but not for those reared at 30 °C. Males showed a significantly stronger preference for outbred females reared at 26 °C than for females from any of the other 3 groups. Inbred females that developed at 26 °C and inbred or outbred females that developed at 30 °C did not differ in their attractiveness (Table 1; Table S1aii). This is not due to the size differences between inbred and outbred females at different temperatures (see Table S5).

**Figure 3:**
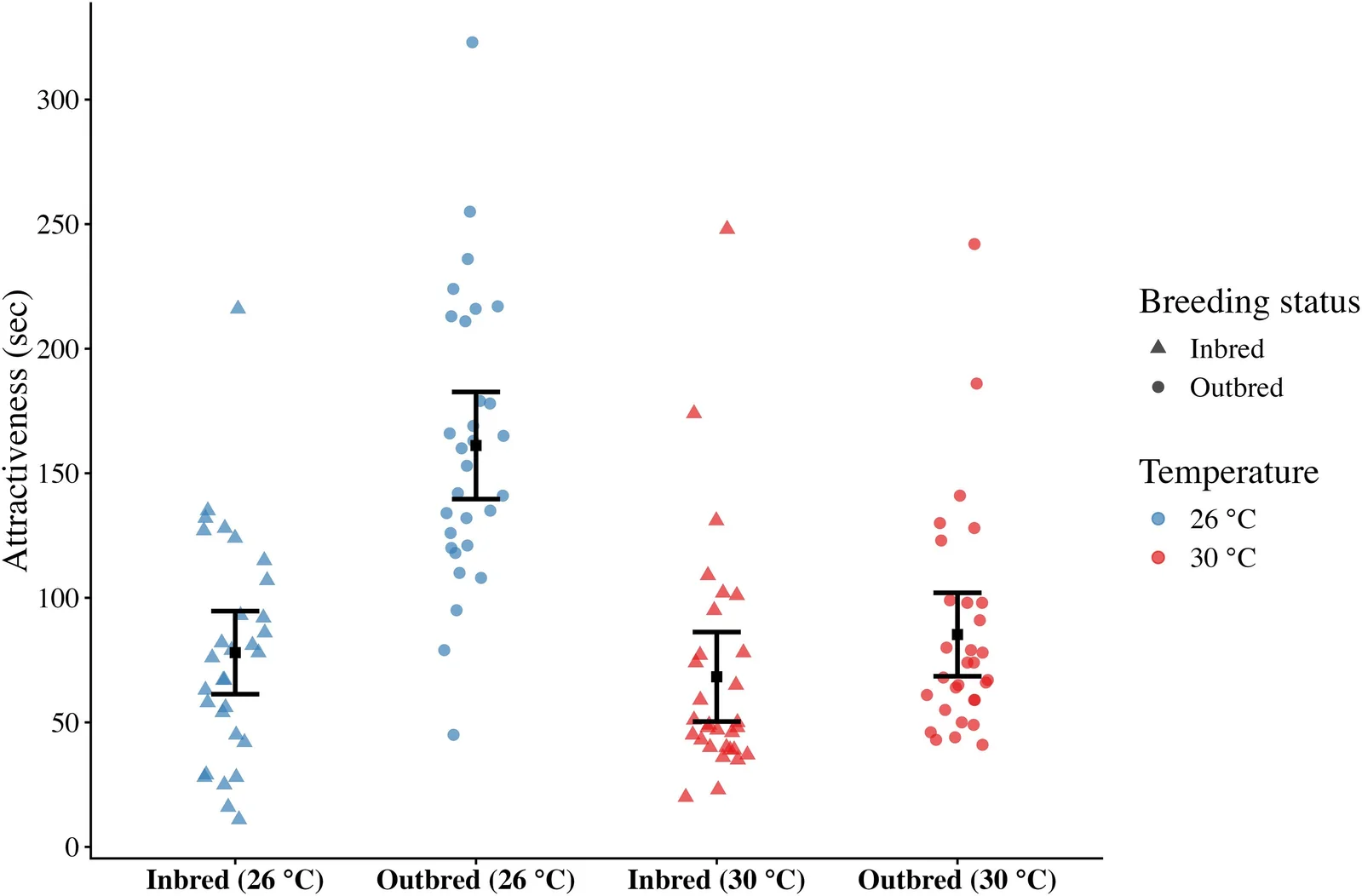
Effects of being inbred and developing at different temperatures on female attractiveness. Each data point represents an individual female (triangles = inbred, circles = outbred, blue = 26 °C, red = 30 °C). Attractiveness was measured as the total time (in seconds) males spent near females. Black squares indicate the mean, and error bars show 95% confidence intervals (CIs).

**Table 1:** Final model outputs for the effects of being inbred and developmental temperature on sexual and reproductive traits of female guppies.

Responses	Predictor	Estimate	SE	$\chi_{1}^{2}$	*P*
Attractiveness (power transformed)	Intercept (outbred, 26 °C)	2.280	0.033	…	
	**Breeding (inbred)**	**−0**.**286**	**0**.**043**	**43**.**261**	**<0.001**
	**Temperature (30 °C)**	**−0**.**241**	**0**.**042**	**33**.**214**	**<0.001**
	Female age difference (mean centered within trial)	0.003	0.002	1.639	0.201
	Female size difference (mean centered within trial)	−0.113	0.116	0.950	0.330
	**Breeding (inbred): Temperature (30 °C)**	**0**.**201**	**0**.**059**	**11**.**635**	**0**.**001**
No. of egg (power transformed)	Intercept (outbred, 26 °C)	0.490	0.034	…	
	Breeding (inbred)	0.011	0.008	2.089	0.148
	Temperature (30 °C)	0.003	0.008	0.183	0.669
	Female age	1.2E-04	3.74E-04	0.101	0.750
	**Female size (standardized)**	**−0**.**027**	**0**.**004**	**38**.**263**	**<0.001**
Total egg mass	Intercept (outbred, 26 °C)	21.445	6.714	…	
	Breeding (inbred)	−0.933	1.490	0.392	0.531
	Temperature (30 °C)	0.148	1.390	0.011	0.915
	Female age	0.070	0.075	0.879	0.349
	**Female size (standardized)**	**6**.**644**	**0**.**861**	**59**.**507**	**<0.001**
Egg diameter (power transformed)	Intercept (outbred, 26 °C)	5.075	1.064	…	
	**Breeding (inbred)**	**−1**.**525**	**0**.**259**	**34**.**770**	**<0.001**
	**Temperature (30 °C)**	**−1**.**291**	**0**.**251**	**26**.**511**	**<0.001**
	**Female age**	**0**.**031**	**0**.**012**	**7**.**104**	**0**.**008**
	Female size (standardized)	0.263	0.138	3.620	0.057

Attractiveness, egg number, and average egg diameter were power-transformed before analysis using *λ* = 0.164, *λ* = −0.274, and *λ* = 3.243, respectively. The table presents model estimates (Estimate), standard errors (SE), chi-square test statistics ($\chi_{1}^{2}$), and *P*-values (*P*). Bold parameters represent significant results.

### Egg traits

None of the 3 egg traits (fecundity, total egg mass, and egg size) were influenced by an interaction between inbreeding and developmental temperature (Table 1).

Neither being inbred nor developing at different temperatures influenced female fecundity or total egg mass. However, larger females produced significantly more eggs, with a significantly greater total egg mass (Figures S1 and S2). Female age had no effect on either trait, but older females have a significantly greater total egg mass if female size was excluded from the analysis (see Supplementary Materials). This is presumably because female age and size were correlated.

In contrast, inbred females and females that developed at 30 °C produced significantly smaller eggs (Fig. 4). Additionally, younger females had significantly smaller eggs, irrespective of whether or not female size was excluded from the analysis (Figure S3). There was a nonsignificant trend (*P* = 0.057) for larger females to produce bigger eggs (Table 1).

**Figure 4:**
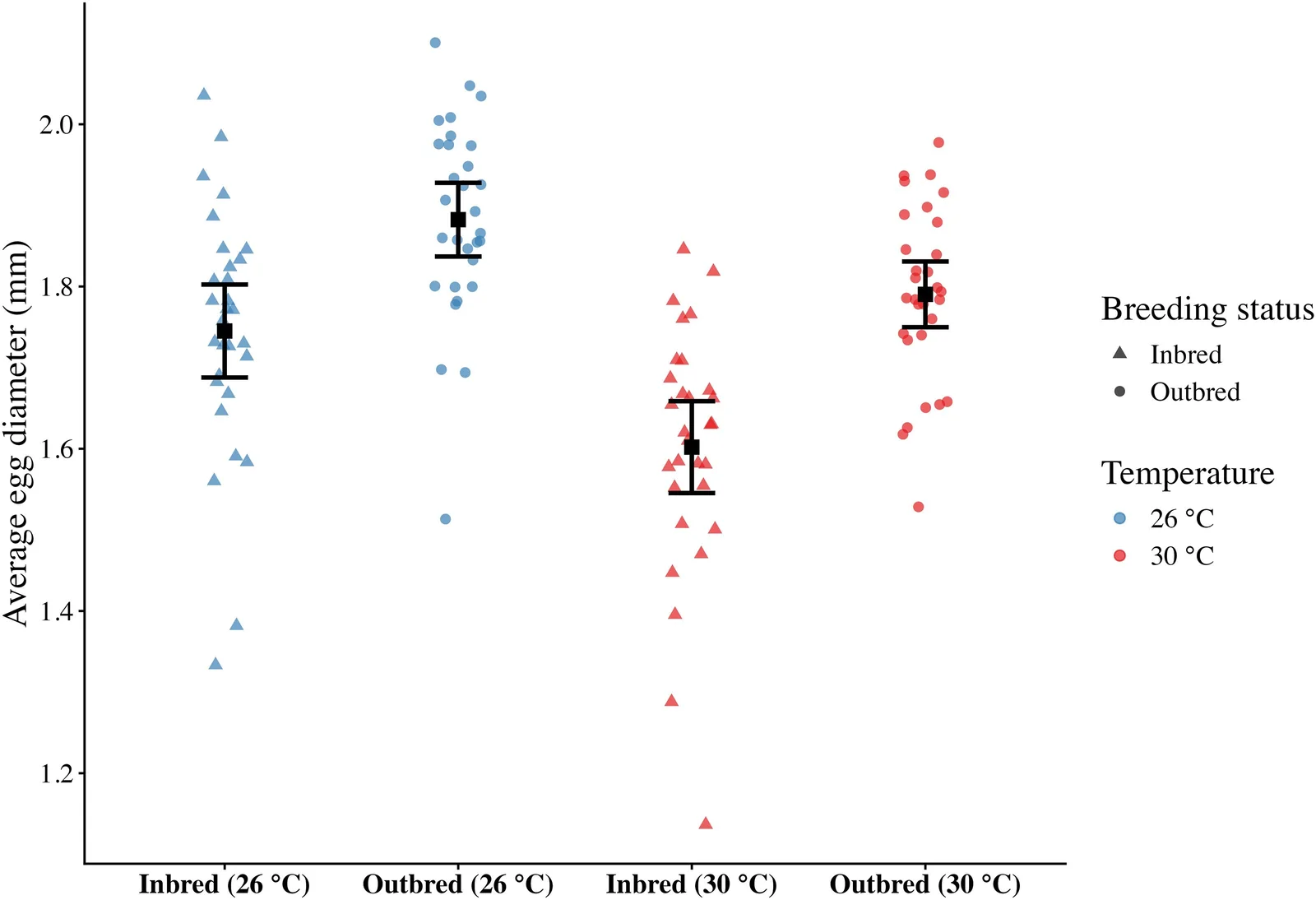
Effects of being inbred and developing at different temperatures on egg diameter. Each data point represents the average diameter of 5 randomly sampled eggs per female (triangles = inbred, circles = outbred, blue = 26 °C, red = 30 °C). Black squares indicate the mean, and error bars show 95% confidence intervals (CIs).

## Discussion

We investigated how inbreeding and elevated developmental temperature affect the sexual and reproductive traits of female guppies (attractiveness measured as male association time, fecundity, total egg mass, and average egg diameter), and whether these 2 factors act independently or interact. Our key findings were 3-fold. First, inbreeding and developmental temperature interacted to affect female attractiveness: males preferred outbred females when females developed at 26 °C, but this preference disappeared when females developed at 30 °C. Second, inbreeding and developmental temperature each independently reduced egg diameter. Third, neither factor affected fecundity or total egg mass. Below, we consider explanations for these findings and their implications for sexual selection in changing environments.

### Effect of inbreeding and developmental temperature on female attractiveness

Male mate choice occurs in many taxa (Bonduriansky 2001; Edward and Chapman 2011). It is generally assumed that males choose which females to court or pursue based on cues that indicate female fecundity, and—in rare cases—their genetic quality (Sandvik et al. 2000). Evidence that animals avoid inbreeding during precopulatory mate choice is mixed, and a recent meta-analysis suggests that active inbreeding avoidance is not the general pattern across animals (de Boer et al. 2021). Indeed, several studies in guppies report no effect of inbreeding on female mate choice (Viken et al. 2006; Pitcher et al. 2008; Guevara-Fiore et al. 2010b). However, we found that male guppies strongly preferred outbred over inbred females when females developed at 26 °C. There is far less evidence that inbreeding makes females less attractive to potential mates (Pölkki et al. 2012). A partial explanation—which we critically evaluate below—for the higher attractiveness of the outbred females in our study is that they were slightly larger than the inbred females (see Figure S4). Prior studies have shown that male poeciliids, including guppies, tend to prefer larger females in dichotomous mate choice tests (Schlupp and Jia 2018). For example, in *Limia perugiae*, a related livebearer, males consistently chose larger females (Powell and Schlupp 2024); and in guppies, some studies show that males make a greater mating effort when courting larger females (eg Herdman et al. 2004), presumably because larger females tend to carry more eggs. It should be noted, however, that other studies fail to detect a male preference for larger females in guppies, and this preference is also affected by social experience (Jordan and Brooks 2012; Auld et al. 2017; Makowicz et al. 2010). Nonetheless, because there are no studies showing preferences for *smaller* females the general trend is towards males preferring larger females.

Using the body size difference between inbred and outbred females to explain why inbred females were less attractive (at least when fish develop at 26 °C) aligns with evidence from earlier studies that inbreeding tends to reduce female body size in guppies (Van Oosterhout et al. 2007; Chung et al. 2024a). Male guppies might gain a direct fitness benefit by preferring outbred and larger (hence potentially more fecund) females (Schlupp and Jia 2018). There are, however, 2 key facts that undermine the explanation that males preferred outbred females because they were larger. First, in our mate choice experiment we used a random sub-sample of 120 of 1,016 females. In the full sample there was no significant difference in the size at maturity of inbred and outbred females (Mahmud-Al-Hasan et al. 2025a). Second, in our analysis we statistically controlled for differences in body size (mean centered within trial) among the 4 females per trial (Table 1), but there was still an effect of female inbreeding (moderated by temperature) on male mate choice. In combination, these 2 facts suggest that body size alone does not explain why inbred females were, on average, less attractive in our study. It is, of course, possible that inbred and outbred females differ in other traits that indicate to males that inbred females are inferior mates.

It is important to note that developmental temperature moderated the effect of inbreeding on female attractiveness in our study. Males preferred outbred females when females developed at 26 °C, but not when females developed at 30 °C. Under normal conditions, outbred females were more attractive, but this inbreeding depression was masked when females experienced thermal stress during development. One possibility is that an elevated temperature might impose physiological costs on females, regardless of their inbreeding status, that lower their body condition and potentially reduce phenotypic differences between inbred and outbred females. This is consistent with the smaller eggs produced by females reared at 30 °C (see below). Intriguingly, however, there is good evidence—albeit from nonsexual traits—that inbreeding depression is magnified, rather than reduced, when animals develop in a stressful environment (Fox and Reed 2011). As such, our findings run counter to the wider trend. In our companion study, inbreeding and temperature also did not interact to affect male attractiveness, and their effects were purely additive (Mahmud-Al-Hasan et al. 2026a). One explanation for our current finding is that while the effect of inbreeding might be greater when there is thermal stress, if there is a reduction in the absolute expression of the cues expressed by females that are used by males then it might be harder to detect differences between females because of the psychophysics of mate choice (eg, a 10% size difference is easier to detect between larger than smaller objects; Cohen 1984). Importantly, this mechanism reduces males' ability to detect differences between females, rather than reducing the underlying effect of inbreeding itself—which could be why egg diameter still shows additive effects of inbreeding and temperature. More generally, there is broad evidence that greater environmental stress reduces the reliability of sexual signals, which reduces the efficacy of mate choice (Candolin and Heuschele 2008; Candolin 2019; Coomes et al. 2019). For example, in pipefish, shifts in temperature can change the strength of mating preferences (Silva et al. 2007). Decreased signal reliability might also explain why male guppies were less discriminating against inbred females when females developed at 30 °C.

### Effect on reproductive traits

Inbreeding had a significant negative effect on only one of the 3 female reproductive traits that we measured. Inbred females produced smaller eggs. In teleost fishes, egg quality depends on maternal vitellogenesis and energy transfer to the oocyte. Reduced maternal condition or compromised follicle development can yield eggs with less yolk that tend to be smaller (Lubzens et al. 2010; Reading et al. 2018). Because egg size in fishes is positively associated with initial offspring size and early performance, even a moderate reduction in egg size can translate into lower offspring quality or reduced early survival rates (Dial et al. 2016; Ponce de León et al. 2011). In contrast to egg size, we did not detect significant inbreeding depression for fecundity or total egg mass suggesting that total reproductive investment does not differ between inbred and outbred females. We therefore found only weak overall evidence that inbreeding lowers fitness-related reproductive traits, despite this being widely reported in many taxa (Crnokrak and Roff 1999; Keller and Waller 2002; although there are many counter-examples such as Mahmud-Al-Hasan et al. 2025b, 2026a). There was also no interaction between inbreeding and developmental temperature for any of the 3 traits that we measured. If we assume that higher temperatures increase stress then our findings are again inconsistent with general evidence that inbreeding depression is stronger when there is greater environmental stress (O’Grady et al. 2006; Fox and Reed 2011).

An elevated developmental temperature had a very similar effect to that of inbreeding on female reproduction: females raised in warmer water produced significantly smaller eggs, but there was no effect on the number of eggs or total egg mass. Our findings fit with well-documented patterns in ectotherms: higher developmental temperatures speed up growth and metabolism but lead to smaller adult size (the “temperature–size rule”; Angilletta and Dunham 2003; Angilletta et al. 2004; Kozłowski et al. 2026). Guppies partly follow this rule. In the full set of females that we subsampled for the current study those raised at 30 °C matured significantly sooner than those raised at 26 °C but they did not differ in their size at maturation (Mahmud-Al-Hasan et al. 2025a). Since females developing in different temperature did not differ in size, and maternal size did not significantly predict egg diameter in our analysis (*χ*^2^ = 3.62, *P* = 0.057), the smaller eggs produced at 30 °C are unlikely to be a downstream consequence of reduced maternal size or body condition. Instead, elevated developmental temperature may have directly affected maternal provisioning—for example through effects on oogenesis and vitellogenesis—independently of female body size.

It is intriguing that we did not detect an effect of developmental temperature on female fecundity or total egg mass. In many fishes, elevated temperature triggers a classic egg size—fecundity tradeoff, with mothers producing more, but smaller, offspring (Smith and Fretwell 1974; Chambers and Leggett 1996; Kamler 2005). Our finding for guppies differs from this pattern. The uncoupling of egg size and number suggests there are physiological limits on reproductive plasticity, perhaps reflecting physiological constraints on resource allocation during oogenesis (Van Der Kraak and Pankhurst 1997; Pankhurst and Munday 2011). There is evidence in guppies that brood size and fry survival drop sharply when water temperature is above ∼28 °C; and that females kept at ≥29 °C often fail to produce offspring, while those kept at 21 to 26 °C produce the most offspring (Karayücel et al. 2008; but see Breckels and Neff 2013). These results suggest that our control temperature of 26 °C (water temperature = 24.4 °C; see Materials and methods) is in the optimal thermal range for guppy reproduction. While females reared at 30 °C (water temperature = 27.8 °C) likely suffered mild but chronic thermal stress; reproduction still occurred, although smaller eggs indicate reduced resource allocation per offspring.

## Conclusion

Inbreeding and higher developmental temperature jointly affected sexual selection on females. Outbred females raised at 26 °C were more attractive than those that developed at 30 °C, but only if females were outbred. The effect of inbreeding therefore depended on the developmental environment. In contrast, inbreeding and developmental temperature independently reduced egg size, but neither factor affected fecundity or total egg mass. More broadly, our findings highlight the role of genotype–environment interactions and suggest that climate warming might alter the impact of inbreeding. It is, however, worth noting that: (i) male mate choice is unlikely to affect female reproductive success in guppies (ie, all females will still acquire mates), although male choice could still lead to genetic evolution if patterns of mating affect parentage which then affects offspring survival (ie who breeds with whom matters as some males are better sires); and (ii) inbreeding did not affect fecundity. It therefore remains an open question as to the extent to which inbreeding will affect population persistence in the wild. Future work should quantify female reproductive output across a broader thermal range and assess whether males also adjust their mate choice based on the ambient temperature or the temperature at which they developed. Understanding the physiological mechanisms that underlie the observed reduction in egg size at 30 °C will also be important to predict the effects, if any, on population persistence from the combined effects of inbreeding and global warming.

## Supplementary Material

arag101_Supplementary_Data

## Data Availability

Analyses reported in this article can be reproduced using the data provided by Mahmud-Al-Hasan et al. (2026b).
