# Supplementary material for: The effect of developmental temperature and inbreeding on sexual and reproductive traits of female guppies
Source: Behav Ecol. 2026 Aug 26;37(6):arag101. doi: 10.1093/beheco/arag101 (PMC13641171; doi:10.1093/beheco/arag101)
Supplement: Supplementary file 1 [file arag101_supplementary_data.docx]

**Supplementary Materials**

**Effect of temperature during development on sexual and reproductive traits of inbred and outbred female guppies (*Poecilia reticulata*)**

***Statistical outputs from models with or without interactions. If left unstated the error distribution was Gaussian.***

**Table S1: Effect of inbreeding and developmental temperature on female attractiveness**

(a) Without the covariate of female size difference
i) Model with the interaction

|  | **Estimate** | ***SE*** | **χ²1** | ***P*** |
| --- | --- | --- | --- | --- |
| Intercept (outbred, 26°C) | 2.274 | 0.032 | 4936.615 | **<0.001** |
| Breeding (inbred) | -0.278 | 0.043 | 42.402 | **<0.001** |
| Temperature (30°C) | -0.237 | 0.042 | 32.406 | **<0.001** |
| Female age difference | 0.002 | 0.002 | 1.093 | 0.296 |
| Breeding (inbred): Temperature (30°C) | 0.198 | 0.059 | 11.361 | 0.001 |

ii) Pairwise comparisons

| Contrast | **Estimate** | ***SE*** | df | *t* | ***P*** |
| --- | --- | --- | --- | --- | --- |
| Outbred 26°C - Inbred 26°C | 0.278 | 0.044 | 85.4 | 6.375 | **<0.001** |
| Outbred 26°C - Outbred 30°C | 0.237 | 0.042 | 80.6 | 5.693 | **<0.001** |
| Outbred 26°C - Inbred 30°C | 0.316 | 0.044 | 85.5 | 7.173 | **<0.001** |
| Inbred 26°C - Outbred 30°C | -0.041 | 0.044 | 85.4 | -0.944 | 0.781 |
| Inbred 26°C - Inbred 30°C | 0.038 | 0.042 | 80.6 | 0.922 | 0.793 |
| Outbred 30°C - Inbred 30°C | 0.079 | 0.044 | 85.5 | 1.804 | 0.279 |

| Random effect | Variance | *SD* | Number of groups |
| --- | --- | --- | --- |
| Block ID | 0 | 0 | 37 |
| Father family ID | 0.001 | 0.036 | 30 |
| Choice trial male ID | 0.003 | 0.058 | 30 |
| Residual | 0.026 | 0.161 |  |

(b) Including female size difference as an additional covariate in the model
i) Model with the interaction

|  | **Estimate** | ***SE*** | **χ²_1_** | ***P*** |
| --- | --- | --- | --- | --- |
| Intercept (outbred, 26°C) | 2.280 | 0.033 | 4812.802 | **< 0.001** |
| Breeding (inbred) | -0.286 | 0.043 | 43.261 | **< 0.001** |
| Temperature (30°C) | -0.241 | 0.042 | 33.214 | **< 0.001** |
| Female age difference | 0.003 | 0.002 | 1.639 | 0.201 |
| Female size difference | -0.113 | 0.116 | 0.950 | 0.330 |
| Breeding (inbred): Temperature (30°C) | 0.201 | 0.059 | 11.635 | **0.001** |

ii) Pairwise comparisons

| Contrast | **Estimate** | ***SE*** | df | *t* | ***P*** |
| --- | --- | --- | --- | --- | --- |
| Outbred 26°C - Inbred 26°C | 0.286 | 0.045 | 83.6 | 6.422 | **<0.001** |
| Outbred 26°C - Outbred 30°C | 0.241 | 0.042 | 79.8 | 5.762 | **<0.001** |
| Outbred 26°C - Inbred 30°C | 0.326 | 0.045 | 83.4 | 7.177 | **<0.001** |
| Inbred 26°C - Outbred 30°C | -0.045 | 0.044 | 84 | -1.018 | 0.739 |
| Inbred 26°C - Inbred 30°C | 0.040 | 0.042 | 79.8 | 0.963 | 0.771 |
| Outbred 30°C - Inbred 30°C | 0.085 | 0.045 | 83.9 | 1.903 | 0.235 |

| Random effect | Variance | *SD* | Number of groups |
| --- | --- | --- | --- |
| Block ID | 0 | 0 | 37 |
| Father family ID | 0.002 | 0.046 | 30 |
| Choice trial male ID | 0.003 | 0.051 | 30 |
| Residual | 0.026 | 0.161 |  |

**Table S2: Effect of inbreeding and developmental temperature on female fecundity**

(a) Without the covariate of female size
i) Model with the interaction

|  | **Estimate** | ***SE*** | **χ²_1_** | ***P*** |
| --- | --- | --- | --- | --- |
| Intercept (outbred, 26°C) | 0.554 | 0.036 | 240.022 | **< 0.001** |
| Breeding (inbred) | 0.020 | 0.013 | 2.528 | 0.112 |
| Temperature (30°C) | 0.008 | 0.012 | 0.438 | 0.508 |
| Female age | -0.001 | 0 | 3.133 | 0.077 |
| Breeding (inbred): Temperature (30°C) | -0.003 | 0.017 | 0.025 | 0.873 |

ii) Final model without the non-significant interaction

|  | **Estimate** | ***SE*** | **χ²_1_** | ***P*** |
| --- | --- | --- | --- | --- |
| Intercept (outbred, 26°C) | 0.554 | 0.035 |  |  |
| Breeding (Inbred) | 0.019 | 0.009 | 4.290 | **0.038** |
| Temperature (30°C) | 0.007 | 0.009 | 0.607 | 0.436 |
| Female age | -0.001 | 3.86E-04 | 3.148 | 0.076 |

| Random effect | Variance | *SD* | Number of groups |
| --- | --- | --- | --- |
| Block ID | 0 | 0 | 37 |
| Father family ID | 0 | 0 | 30 |
| Residual | 0.002 | 0.048 |  |

iii) Comparing the model fit (with vs without interaction)

| Model | Df | AIC | BIC | logLik | Deviance | χ² | Df | *P* |
| --- | --- | --- | --- | --- | --- | --- | --- | --- |
| ii | 7 | -377.77 | -358.26 | 195.88 | -391.77 | 0.027 | 1 | 0.871 |
| i | 8 | -375.8 | -353.5 | 195.9 | -391.8 |  |  |  |

(b) Including female size as an additional covariate in the model
i) Model with the interaction

|  | **Estimate** | ***SE*** | **χ²_1_** | ***P*** |
| --- | --- | --- | --- | --- |
| Intercept (outbred, 26°C) | 0.49 | 0.034 | 209.251 | **< 0.001** |
| Breeding (inbred) | 0.011 | 0.011 | 1.038 | 0.308 |
| Temperature (30°C) | 0.003 | 0.011 | 0.080 | 0.777 |
| Female age | 1.1E-04 | 3.75E-04 | 0.1 | 0.752 |
| Female size (standardized) | -0.027 | 0.004 | 37.789 | **< 0.001** |
| Breeding (inbred): Temperature (30°C) | 4E-04 | 0.015 | 0.001 | 0.979 |

ii) Final model without the non-significant interaction

|  | **Estimate** | ***SE*** | **χ²_1_** | ***P*** |
| --- | --- | --- | --- | --- |
| Intercept (outbred, 26°C) | 0.490 | 0.034 |  |  |
| Breeding (inbred) | 0.011 | 0.008 | 2.089 | 0.148 |
| Temperature (30°C) | 0.003 | 0.008 | 0.183 | 0.669 |
| Female age | 1.2E-04 | 3.74E-04 | 0.101 | 0.750 |
| Female size (standardized) | -0.027 | 0.004 | 38.263 | **< 0.001** |

| Random effect | Variance | *SD* | Number of groups |
| --- | --- | --- | --- |
| Block ID | 0 | 0 | 37 |
| Father family ID | 9.42E-05 | 0.01 | 30 |
| Residual | 1.68E-03 | 0.041 |  |

iii) Comparing the model fit (with vs without interaction)

| Model | Df | AIC | BIC | logLik | Deviance | χ² | Df | *P* |
| --- | --- | --- | --- | --- | --- | --- | --- | --- |
| ii | 8 | -409.21 | -386.91 | 212.61 | -425.21 | 7E-04 | 1 | 0.979 |
| i | 9 | -407.22 | -382.13 | 212.61 | -425.22 |  |  |  |

**Table S3: Effect of inbreeding and developmental temperature on total egg mass**

(a) Without the covariate of female size
i) Model with the interaction

|  | **Estimate** | ***SE*** | **χ²_1_** | ***P*** |
| --- | --- | --- | --- | --- |
| Intercept (outbred, 26°C) | 6.327 | 8.070 | 0.615 | 0.433 |
| Breeding (inbred) | -3.144 | 2.436 | 1.666 | 0.197 |
| Temperature (30°C) | -1.067 | 2.378 | 0.201 | 0.654 |
| Female age | 0.263 | 0.088 | 8.920 | **0.003** |
| Breeding (inbred): Temperature (30°C) | 0.627 | 3.364 | 0.035 | 0.852 |

ii) Final model without the non-significant interaction

|  | **Estimate** | ***SE*** | **χ²_1_** | ***P*** |
| --- | --- | --- | --- | --- |
| Intercept (outbred, 26°C) | 6.270 | 8.022 |  |  |
| Breeding (Inbred) | -2.837 | 1.763 | 2.590 | 0.108 |
| Temperature (30°C) | -0.753 | 1.672 | 0.203 | 0.652 |
| Female age | 0.262 | 0.088 | 8.902 | **0.003** |

| Random effect | Variance | *SD* | Number of groups |
| --- | --- | --- | --- |
| Block ID | 10.461 | 3.234 | 37 |
| Father family ID | 9.354 | 3.058 | 30 |
| Residual | 83.794 | 9.154 |  |

iii) Comparing the model fit (with vs without interaction)

| Model | Df | AIC | BIC | logLik | Deviance | χ² | Df | *P* |
| --- | --- | --- | --- | --- | --- | --- | --- | --- |
| ii | 7 | 902.44 | 921.96 | -444.22 | 888.44 | 0.036 | 1 | 0.849 |
| i | 8 | 904.41 | 926.71 | -444.2 | 888.41 |  |  |  |

b) Including female size as an additional covariate in the model
i) Model with the interaction

|  | **Estimate** | ***SE*** | **χ²_1_** | ***P*** |
| --- | --- | --- | --- | --- |
| Intercept (outbred, 26°C) | 21.403 | 6.752 | 10.047 | 0.002 |
| Breeding (inbred) | -0.852 | 2.046 | 0.173 | 0.677 |
| Temperature (30°C) | 0.230 | 1.977 | 0.014 | 0.907 |
| Female age | 0.070 | 0.075 | 0.872 | 0.350 |
| Female size (standardized) | 6.643 | 0.865 | 58.984 | **< 0.001** |
| Breeding (inbred): Temperature (30°C) | -0.164 | 2.789 | 0.004 | 0.953 |

ii)	Final model without the non-significant interaction

|  | **Estimate** | ***SE*** | **χ²_1_** | ***P*** |
| --- | --- | --- | --- | --- |
| Intercept (outbred, 26°C) | 21.445 | 6.714 |  |  |
| Breeding (inbred) | -0.933 | 1.490 | 0.392 | 0.531 |
| Temperature (30°C) | 0.148 | 1.390 | 0.011 | 0.915 |
| Female age | 0.070 | 0.075 | 0.879 | 0.349 |
| Female size (standardized) | 6.644 | 0.861 | 59.507 | **< 0.001** |

| Random effect | Variance | *SD* | Number of groups |
| --- | --- | --- | --- |
| Block ID | 10.52 | 3.244 | 37 |
| Father family ID | 1.28E-06 | 0.001 | 30 |
| Residual | 57.530 | 7.585 |  |

iii) Comparing the model fit (with vs without interaction)

| Model | Df | AIC | BIC | logLik | Deviance | χ² | Df | *P* |
| --- | --- | --- | --- | --- | --- | --- | --- | --- |
| ii | 8 | 854.7 | 877 | -419.35 | 838.7 | 0.004 | 1 | 0.952 |
| i | 9 | 856.69 | 881.78 | -419.35 | 838.69 |  |  |  |

**Table S4: Effect of inbreeding and developmental temperature on egg diameter**

a) Without the covariate of female size
i) Model with the interaction

|  | **Estimate** | ***SE*** | **χ²_1_** | ***P*** |
| --- | --- | --- | --- | --- |
| Intercept (outbred, 26°C) | 4.463 | 1.047 | 18.174 | < 0.001 |
| Breeding (inbred) | -1.470 | 0.357 | 16.960 | **< 0.001** |
| Temperature (30°C) | -1.194 | 0.353 | 11.439 | **< 0.001** |
| Female age | 0.038 | 0.011 | 11.368 | **< 0.001** |
| Breeding (inbred): Temperature (30°C) | -0.264 | 0.500 | 0.280 | 0.597 |

ii)	Final model without the non-significant interaction

|  | **Estimate** | ***SE*** | **χ²_1_** | ***P*** |
| --- | --- | --- | --- | --- |
| Intercept (outbred, 26°C) | 4.523 | 1.040 |  |  |
| Breeding (Inbred) | -1.602 | 0.255 | 39.409 | **< 0.001** |
| Temperature (30°C) | -1.327 | 0.249 | 28.439 | **< 0.001** |
| Female age | 0.038 | 0.011 | 11.452 | **< 0.001** |

| Random effect | Variance | *SD* | Number of groups |
| --- | --- | --- | --- |
| Block ID | 0 | 0 | 37 |
| Father family ID | 0.062 | 0.249 | 30 |
| Residual | 1.856 | 1.362 |  |

iii)	comparing the model fit (with vs without interaction)

| Model | Df | AIC | BIC | logLik | Deviance | χ² | Df | *P* |
| --- | --- | --- | --- | --- | --- | --- | --- | --- |
| ii | 7 | 428.39 | 447.9 | -207.19 | 414.39 | 0.29 | 1 | 0.59 |
| i | 8 | 430.1 | 452.4 | -207.05 | 414.1 |  |  |  |

b) Including female size as an additional covariate in the model
i) Model with the interaction

|  | **Estimate** | ***SE*** | **χ²_1_** | ***P*** |
| --- | --- | --- | --- | --- |
| Intercept (outbred, 26°C) | 5.020 | 1.071 | 21.971 | **< 0.001** |
| Breeding (inbred) | -1.377 | 0.361 | 14.565 | **< 0.001** |
| Temperature (30°C) | -1.142 | 0.355 | 10.331 | **0.001** |
| Female age | 0.031 | 0.012 | 6.957 | **0.008** |
| Female size (standardized) | 0.266 | 0.139 | 3.670 | 0.055 |
| Breeding (inbred): Temperature (30°C) | -0.296 | 0.502 | 0.347 | 0.556 |

ii)	Final model without the non-significant interaction

|  | **Estimate** | ***SE*** | **χ²_1_** | ***P*** |
| --- | --- | --- | --- | --- |
| Intercept (outbred, 26°C) | 5.075 | 1.064 |  |  |
| Breeding (inbred) | -1.525 | 0.259 | 34.770 | **< 0.001** |
| Temperature (30°C) | -1.291 | 0.251 | 26.511 | **< 0.001** |
| Female age | 0.031 | 0.012 | 7.104 | **0.008** |
| Female size (standardized) | 0.263 | 0.138 | 3.620 | 0.057 |

| Random effect | Variance | *SD* | Number of groups |
| --- | --- | --- | --- |
| Block ID | 0 | 0 | 37 |
| Father family ID | 0 | 0 | 30 |
| Residual | 1.873 | 1.369 |  |

iii)	Comparing the model fit (with vs without interaction)

| Model | Df | AIC | BIC | logLik | Deviance | χ² | Df | *P* |
| --- | --- | --- | --- | --- | --- | --- | --- | --- |
| ii | 8 | 426.770 | 449.070 | -205.38 | 410.77 | 0.365 | 1 | 0.546 |
| i | 9 | 428.410 | 453.490 | -205.2 | 410.41 |  |  |  |

**Figure S1:** Relationship between female body size and fecundity in guppies. Larger females produced more eggs (Pearson’s *r* = 0.50, p < 0.001, N = 120). The solid line represents the fitted linear regression; the shaded area shows the 95% confidence interval.

**Figure S2:** Relationship between female body size and total egg mass in guppies. A significant positive correlation was observed, indicating that larger females tended to produce heavier clutches (Pearson’s *r* = 0.65, p < 0.001, N = 120). The solid line represents the fitted linear regression, and the shaded area shows the 95% confidence interval.

**Figure S3:** Relationship between female age and egg diameter in guppies. Older females produced larger eggs (Pearson’s *r* = 0.32, p < 0.001, N = 120). The solid line represents the fitted regression, with the shaded area indicating the 95% confidence interval.

**Figure S4:** Effect of inbreeding on female body size in guppies (*Poecilia reticulata*). Each point represents the body length of an individual female from either inbred (red) or outbred (blue) treatments. Black squares indicate group means (± 95% confidence intervals). A significant effect of inbreeding was detected (χ² = 4.18, P = 0.04, N = 60 outbred and 60 inbred). After excluding the very small female (size = 12.48 mm), there was no longer a significant difference in size between our inbred and outbred females (P = 0.06). This mature female was unusually small (see image below). Therefore, this was not a data entry error.

**Table S5: Effect of inbreeding and developmental temperature on female body size (standard length)**

*Model with the interaction*

|  | **Estimate** | **SE** | **χ²₁** | **P** |
| --- | --- | --- | --- | --- |
| Intercept (outbred, 26°C) | 18.384 | 1.450 | 160.719 | <0.001 |
| Breeding (inbred) | −0.699 | 0.392 | 3.181 | 0.074 |
| Temperature (30°C) | −0.391 | 0.382 | 1.050 | 0.306 |
| Age on trial date | 0.056 | 0.016 | 12.603 | <0.001 |
| Breeding (inbred) × Temperature (30°C) | 0.237 | 0.540 | 0.192 | 0.661 |

**Final model without the non-significant interaction**

|  | **Estimate** | **SE** | **χ²₁** | **P** |
| --- | --- | --- | --- | --- |
| Intercept (outbred, 26°C) | 18.357 | 1.443 | — | — |
| Breeding (inbred) | −0.583 | 0.285 | 4.183 | 0.041 |
| Temperature (30°C) | −0.273 | 0.269 | 1.033 | 0.310 |
| Age on trial date | 0.056 | 0.016 | 12.524 | <0.001 |
